# Biomechanical Compensation Patterns Across Different Phases of Side-Cutting Following Anterior Cruciate Ligament Reconstruction

**DOI:** 10.3390/bioengineering12121280

**Published:** 2025-11-21

**Authors:** Mingxuan Gao, Xialin Ge, Yiming Tao, Longting Suo, Shuang Ren, Yingfang Ao

**Affiliations:** 1 Tianjin Key Laboratory of Exercise Physiology and Sports Medicine, Institute of Sport, Exercise & Health, Tianjin University of Sport, Tianjin 300381, China; gmx15831545021@163.com (M.G.); gxl990829@163.com (X.G.); 18596128002@163.com (Y.T.); 19025280592@163.com (L.S.); 2 Department of Sports Medicine, Peking University Third Hospital, Institute of Sports Medicine of Peking University, Beijing 100191, China; 3 Beijing Key Laboratory of Sports Injuries, Beijing 100191, China; 4 Engineering Research Center of Sports Trauma Treatment Technology and Devices, Ministry of Education, Beijing 100191, China

**Keywords:** anterior cruciate ligament reconstruction, side-cutting, phase specific, compensatory mechanisms

## Abstract

(1) Background: Anterior cruciate ligament reconstruction (ACLR) alters lower-limb biomechanics. While gait and running are well-studied, the multi-phase side-cutting remains poorly understood, particularly regarding phase-specific adaptations after ACLR. (2) Methods: Thirty-four patients (19 male, 15 female) at nine months post-ACLR participated. Biomechanical data during side-cutting were collected using synchronized motion capture and force platforms. Knee joint kinematics and kinetics were analyzed over three phases: initial contact-deceleration, stance pivot, and push-off. (3) Results: During the initial contact-deceleration, the reconstructed limb exhibited greater knee external rotation at the first posterior ground reaction force (pGRF) peak (8.5° vs. 6.3°, *p* = 0.021), and reduced knee flexion (43.2° vs. 47.3°, *p* < 0.001) with a lower extension moment at the second pGRF peak (0.10 vs. 0.14 BW·BH; *p* < 0.001). The stance pivot phase was marked by significantly lower knee flexion (*p* = 0.001), extension moment (*p* < 0.001), and medial/vertical GRFs on the reconstructed side (0.49 vs. 0.52 BW, *p* = 0.029; 1.98 vs. 2.10 BW, *p* = 0.012). During the push-off, the involved limb demonstrated a significantly lower extension moment (0.008 vs. 0.014 BW·BH, *p* = 0.037) and anterior GRF (0.20 vs. 0.23 BW, *p* = 0.010). (4) Conclusions: This study proposes a three-phase compensation model for side-cutting: “rotational instability” at initial contact, “protective unloading” during the stance pivot phase, and “force-generation deficit” at push-off. This three-phase framework provides a new paradigm for evaluating dynamic knee function after ACLR and guiding phase-specific rehabilitation.

## 1. Introduction

Anterior cruciate ligament reconstruction (ACLR) is a standard surgical procedure aimed at restoring knee joint stability and potentially minimizing the risk of future degenerative changes [1]. However, postoperative recovery outcomes demonstrate significant variability, with approximately 65% of patients achieving return-to-sport at preinjury levels and only 55% returning to competitive play [2]. Notably, the overall reinjury rate remains high at 15% [3], a problem closely linked to high-risk maneuvers like side-cutting, which account for approximately 70% of both primary [4] and secondary ACL injuries [5]. These movements require athletes to rapidly decelerate, change direction, and accelerate again within a constrained space [6,7], during which the lower extremities sustain substantial impact loads that significantly increase joint injury risk [8].

Previous studies have identified marked biomechanical abnormalities [9,10] and neuromuscular adaptation [11] after ACLR, which can persist for years post-surgery [12]. However, the current literature mainly focuses on low-risk movements, such as gait and running [13,14], or isolated biomechanical parameters during side-cutting [15]. A comprehensive, phase-specific analysis of the entire side-cutting sequence remains lacking.

Given that the 9-month postoperative phase is a critical window for functional recovery and return to sport [16], a systematic analysis of the biomechanics during side-cutting is essential. This is crucial for uncovering persistent neuromuscular control deficits that may not be apparent during basic athletic tasks [17]. To address this gap, this study proposes a novel three-phase compensatory framework to characterize lower-extremity biomechanics during side-cutting after ACLR. This framework aims to advance our understanding of dynamic compensation and provide improved clinical guidance for rehabilitation.

This framework delineates the side-cutting into three distinct phases: the initial contact-deceleration phase [7,18] (generating posterior ground reaction forces, pGRF), the stance pivot phase [18,19] (producing medial ground reaction forces, mGRF), and the push-off phase [20] (creating anterior propulsion forces, aGRF). We hypothesize that, compared to the contralateral limb, the reconstructed limb would exhibit phase-specific asymmetries during side-cutting: greater external rotation at the initial contact-deceleration phase, reduced loading (lower GRFs) during the stance pivot, and decreased propulsion force generation at push-off. Through a systematic analysis of knee joint kinematics and kinetics across these three-phases, this study aims to establish the proposed framework, thereby providing a more nuanced and clinically actionable understanding of dynamic dysfunction after ACLR.

## 2. Materials and Methods

### 2.1. Participants

**Figure 1:**
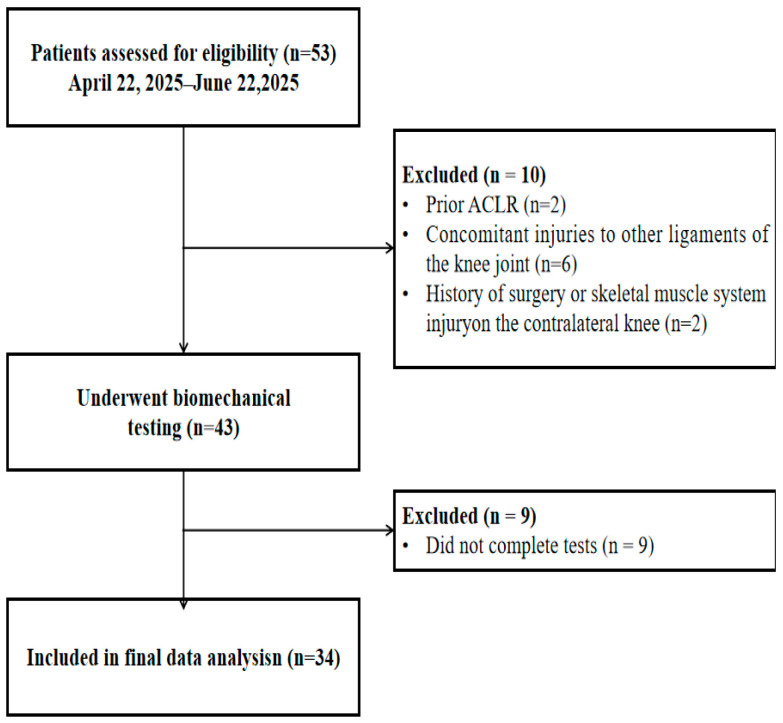
A flowchart describing the inclusion and exclusion of participants. ACLR, anterior cruciate ligament reconstruction.

Participants were recruited from patients attending the outpatient department for follow-up. Inclusion criteria were: (1) 9 months after unilateral ACL reconstruction with an autologous hamstring tendon graft; (2) no other ligament injuries or comorbidities affecting lower limb biomechanics; (3) preinjury Tegner Activity Scale [21] score > 5. Moreover, all ACLR participants adhered to a standardized home-based rehabilitation protocol. At the time of testing, patients had regained a normal joint range of motion and had no other restrictions. This research has been approved by the hospital’s ethics committee (No. M20250039). All participants read and signed an informed consent form before data collection. The enrollment procedure is shown in Figure 1.

### 2.2. Testing Protocol

For biomechanical testing, participants performed side-cutting while wearing their athletic shoes, shorts, and tight-fitting upper-body clothing. A modified Plug-in-Gait model was used, with retroreflective markers placed on the following anatomical landmarks: the anterior superior iliac spine (Asis), posterior superior iliac spine (Psis), mid-thigh (50% of thigh length), distal lateral third of the thigh, lateral and medial femoral condyles, proximal and distal thirds of the tibia, lateral distal third of the tibia, lateral and medial malleoli, calcaneus, and the first, second, and fifth metatarsophalangeal joints. Eligible participants were recruited and asked to complete the Tegner Activity Scale to assess their pre-injury activity levels. Subsequently, they completed the 2000 International Knee Documentation Committee (IKDC) Subjective Knee Form [22] and Lysholm Knee Scoring Scale to [23] evaluate knee-related symptoms and functional limitations.

### 2.3. Data Collection

Three-dimensional kinematic data were captured using a 12-camera infrared motion capture system (Vicon Motion Systems Ltd., Yarnton, UK; Version 1.8.5) at 100 Hz. Kinetic parameters were collected with two force platforms (Advanced Mechanical Technology, Watertown, MA, USA; Version BP400600), which sampled at 1000 Hz. Kinematic and kinetic data were synchronized using an analog-to-digital converter (AMTI GEN5, Advanced Mechanical Technology, USA). Before biomechanical testing, a static calibration trial was acquired for each participant in the standing position. Participants performed 10–15 min of warm-up, followed by a demonstration and practice of the side-cutting task. During formal testing, participants were instructed to: sprint at maximal velocity, then execute a 45-degree side-cutting on the force platform at maximum speed, followed immediately by resuming maximal sprinting [24]. To ensure proper foot placement on the center of the force platform and to minimize variability, participants completed at least three successful practice trials before data collection. Five trials were performed per participant, with a minimum of three valid trials retained for analysis.

### 2.4. Data Reduction

The raw motion capture data were processed using Vicon Nexus software (Vicon Motion Systems Ltd., UK; Version 1.8.5) for reflective marker identification and trajectory reconstruction, followed by smoothing of all three-dimensional marker coordinates with a fourth-order Butterworth low-pass filter at a 10 Hz cutoff frequency before being exported in C3D format. These processed C3D files were subsequently imported into Visual 3D software (C-Motion, Germantown, MD, USA; Version 6.01.0), where segmental coordinate systems were established for the thigh and shank segments, enabling the computation of kinematic and kinetic parameters. All reported moments represent internal moments throughout this study.

Ground reaction forces include anterior (+)/posterior (−) GRF along the Y-axis, medial (−)/lateral (+) GRF along the X-axis, and vertical GRF along the Z-axis. IC was defined as the first point at which the vGRF exceeded the 10 N threshold. Toe-off (TO) was subsequently determined as the point at which the vGRF dropped below the 10 N threshold following IC. Knee joint angles were calculated as Cardan angles of a distal segment reference frame relative to the proximal segment reference frame in order of (1) flexion–extension, (2) abduction–adduction, and (3) internal–external rotation.

**Figure 2:**
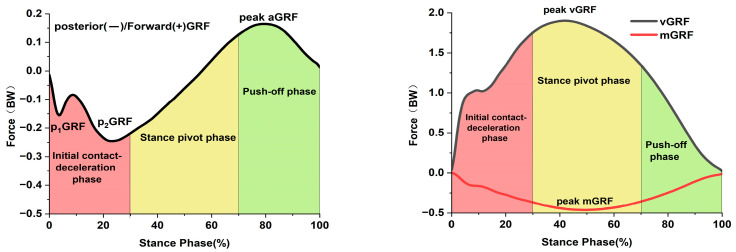
Triaxial ground reaction forces during the stance phase. GRF, ground reaction force; p_1_GRF, first posterior ground reaction force; p_2_GRF, second posterior ground reaction force; vGRF, vertical ground reaction force; mGRF, medial ground reaction force; aGRF, anterior ground reaction force; BW, body weight.

This study identified three typical phases that characterized side-cutting movements: (1) The initial contact-deceleration phase, where the first peak posterior ground reaction force (p_1_GRF) and the second peak posterior ground reaction force (p_2_GRF) are generated [7]; (2) The stance pivot phase, where the peak medial ground reaction force (mGRF) and the peak vertical ground reaction force (vGRF) appear [18]; (3) The push-off phase, where the peak anterior ground reaction force (aGRF) is produced [15]. The kinematic characteristics across these three phases were more systematic than in previous studies that focused solely on pGRF and vGRF (Figure 2). To ensure consistency and objectivity while avoiding subjective bias, a standardized, custom-written script in Visual 3D was used to automatically identify all characteristic GRF peaks. For each participant, the mean value across all valid trials (3 to 5) was calculated and subsequently used for statistical analysis.

This study systematically analyzed the following parameters based on the three-phase division: (1) peak ground reaction forces in different directions and the knee joint angles and moments at the peak values; (2) the maximum knee joint angles and moments throughout the entire support phase. Ground reaction forces are normalized to body weight, denoted by BW; moments are normalized to the product of body weight and height, denoted by BW·BH. The entire support phase of the side-cutting movement is standardized to 101 discrete points, corresponding to 0 to 100% of the support phase.

### 2.5. Statistical Analysis

The Shapiro–Wilk test confirmed that all data were normally distributed. Paired *t*-tests were employed to compare ground reaction forces, knee joint angles, and moments between the reconstructed and contralateral limbs in ACLR participants. The sample-size estimation was based on an expected effect size of Cohen’s dz = 0.80, an alpha level of 0.05 (two-tailed), and a power of 0.80 [10]. G*Power 3.1 indicated that 26 participants would be required under these assumptions. To ensure adequate power and to account for potential dropouts, we enrolled 34 ACLR participants. Continuous data are presented as mean ± standard deviation. All analyses were performed with SPSS (Version 25.0, IBM Corp., Armonk, NY, USA), with statistical significance set at *p* < 0.05. For all statistically significant results, effect sizes were calculated and reported using Cohen’s d. It was classified as follows: no (<0.2), small (≥0.2 and <0.5), medium (≥0.5 and <0.8), and large (≥0.8) effect [25].

## 3. Results

This study enrolled 34 participants who had undergone anterior cruciate ligament (ACL) reconstruction (Table 1).

### 3.1. Maximum Knee Joint Angles and Moments During Side-Cutting

The reconstructed limb demonstrated a significantly lower peak flexion angle (*p* < 0.001, Cohen’s d = 0.85; Table 2), a lower peak extension moment (*p* < 0.001; Cohen’s d = 1.06; Table 2), and a greater peak external rotation angle (*p* = 0.037, Cohen’s d = 0.37; Table 2) compared to the contralateral limb.

### 3.2. Knee Kinematics and Kinetics During the Three Phases of Side-Cutting

The biomechanical analysis revealed significant inter-limb differences across all three phases of the side-cutting task. Table 3 provides a concise summary of all key findings, including knee kinematics, kinetics, and ground reaction forces. The detailed results for each phase are presented below.

#### 3.2.1. Initial Contact-Deceleration Phase

During the initial contact-deceleration phase, no significant inter-limb differences were found in the p_1_GRF and p_2_GRF magnitudes themselves (*p* = 0.430, *p* = 0.280; Table 4). However, at the peak of p_1_GRF, the reconstructed limb exhibited significantly greater external rotation and a reduced extension moment compared to the contralateral limb (*p* = 0.021, Cohen’s d = 0.42; *p* = 0.020, Cohen’s d = 0.42; Table 4). At the peak of p_2_GRF, the reconstructed limb showed a significantly smaller flexion angle, accompanied by reduced extension and abduction moments (*p* < 0.001, Cohen’s d = 0.81; *p* < 0.001, Cohen’s d = 0.79; *p* = 0.038, Cohen’s d = 0.37; Table 4).

#### 3.2.2. Stance Pivot Phase

During the stance pivot phase, the reconstructed limb exhibited significantly attenuated peak mGRF and vGRF (*p* = 0.029, Cohen’s d = 0.39; *p* = 0.012, Cohen’s d = 0.46; Table 5). Concurrently, significantly reduced knee flexion angles (*p* < 0.001, Cohen’s d = 0.67; *p* < 0.001, Cohen’s d = 0.63; Table 5) and extension moments (*p* = 0.037, Cohen’s d = 0.96; *p* = 0.036, Cohen’s d = 0.82; Table 5) were identified at both the mGRF and vGRF peaks. A significantly greater external rotation angle was also observed at the vGRF peak (*p* = 0.036, Cohen’s d = 0.37; Table 5).

#### 3.2.3. Push-Off Phase

During the push-off phase, the reconstructed limb exhibited a significantly attenuated peak aGRF (*p* = 0.010, Cohen’s d = 0.47; Table 6). This phase was also characterized by a significantly reduced knee extension moment at the point of peak aGRF (*p* = 0.037, Cohen’s d = 0.37; Table 6).

## 4. Discussion

The principal contribution of this study is the articulation of a three-phase compensatory framework during side-cutting post-ACLR, which moves beyond previous unidimensional and isolated parameter analyses. Based on this framework, our results reveal a dynamic compensation pattern for the knee joint during side-cutting after ACLR surgery: “rotational instability” upon initial contact-deceleration phase, “protective unloading” during the stance pivot phase, and “force-generation deficit” at push-off phase.

Our analysis of the initial contact-deceleration phase revealed a critical, biphasic compensatory strategy. At the first posterior GRF peak (p_1_GRF), the reconstructed limb exhibited significantly greater external rotation angle alongside a reduced extension moment, a pattern we term “rotational instability”. This aligns with reports that ACL injuries often occur during early deceleration [26], a phase where biomechanical analysis revealed that deceleration initiation generates immediate posterior GRF. Furthermore, existing literature [27] confirms maximal ACL strain occurs at the instant of the first posterior GRF peak (p_1_GRF), while demonstrating lower GRF magnitude and muscular activation compared to the second posterior GRF peak [28]. This phenomenon likely stems from a combination of altered afferent sensory feedback due to the injury and subsequent behavioral motor control compensations [29]. Furthermore, the loss of ligament mechanoreceptors, together with the associated physiological cascade of inflammation and joint effusion, alters afferent input to the central nervous system [30,31]. This disrupted signaling subsequently leads to a series of abnormal biomechanical patterns. Previous studies have found that ACL reconstruction surgery can partially restore anterior tibial translation; however, rotational control remains significantly impaired during high-risk activities [32]. Furthermore, longitudinal studies indicate that persistent abnormal rotational kinematics in reconstructed knees may contribute to progressive cartilage degeneration [33], and over time, this can result in knee osteoarthritis [34].

Subsequently, at the p_2_GRF peak, the limb appears to adopt a stiffening strategy [35] to compensate for this initial instability, evidenced by a significantly reduced flexion angle and diminished extension/abduction moments [15]. This compensatory strategy likely represents a neuromuscular adaptation to rotational control deficits during the initial p_1_GRF phase, where restricted joint range of motion enhances knee stability. However, the reduced knee flexion angle increases the deviation angle of the ACL, amplifying quadriceps-induced anterior pull on the tibia and consequently elevating reinjury risk [36]. Notably, Walsh et al. [37] reported that neuromuscular control training in post-ACLR patients significantly improved landing mechanics, evidenced by increased knee flexion angles and enhanced hamstring activation, which effectively reduced anterior tibial shear forces, consequently decreasing reinjury risk. These findings provide compelling evidence that targeted intervention optimizing landing techniques can modify lower extremity kinetics, offering a critical biomechanical rationale for refining ACL rehabilitation protocols.

During the stance-pivot phase, the reconstructed limb demonstrated significantly lower mGRF and vGRF compared to the contralateral limb, confirming persistent lower extremity loading asymmetry at 9 months post-ACLR, consistent with previous literature [38]. Research indicates [39] that patients shortly after ACLR surgery tend to limit weight-bearing on the affected side to alleviate discomfort. Notably, at the mGRF peak, the reconstructed limb exhibited significantly reduced knee flexion angle and extension moment. These kinematic-kinetic abnormalities were more pronounced at the vGRF peak, collectively supporting the existence of a potential “protective unloading strategy” [40]. This observation is supported by the work of Tayfur et al. [17], who demonstrated persistent muscle strength deficits and neuromuscular alterations after ACLR. In addition, psychological factors represent a key area of focus. Existing research establishes that fear of reinjury correlates with both functional and biomechanical outcomes following ACLR [41]. Future rehabilitation strategies, therefore, should target these domains to improve efficacy and effectively prevent secondary injuries.

During the push-off phase, the reconstructed limb exhibited a significant “force-generation deficit,” characterized by markedly reduced aGRF and knee extension moment compared to the contralateral limb. This deficit is primarily due to ongoing weakness in the quadriceps [28]. These findings support existing evidence showing long-term quadriceps strength deficits after ACLR [42]. The correlation analysis by Ren and Huang [43] confirmed a strong relationship between quadriceps strength asymmetry and knee extension moment asymmetry during gait. Schmitt et al. [44] further reported that athletes with quadriceps weakness exhibited abnormal landing kinematics, underscoring the critical role of lower extremity strength in ACL rehabilitation. These collective findings support implementing phased strength training throughout rehabilitation to address dynamic stability during initial contact sequentially [45], weight-bearing capacity during mid-stance, and propulsive power during push-off.

However, this study has several limitations. First, the lack of long-term follow-up data prevents assessment of whether compensation persists beyond 9 months. In future studies, the collection of longitudinal data at longer time points, such as 12 or 18 months, will be considered to validate whether the compensatory model persists or evolves over time. Second, this study focused exclusively on knee joint biomechanics. Consequently, our findings do not fully capture compensatory mechanisms from a complete kinetic chain perspective. Therefore, investigating the roles of the hip and ankle remains a critical future direction. Third, the absence of a healthy control group, as well as the lack of recorded limb dominance data, limits the interpretation of the observed biomechanical asymmetries. Fourth, due to the sample size limitation, we were unable to analyze the potential influence of sex on the observed compensatory patterns during the side-cutting. Future studies with larger samples are needed to systematically examine sex as a key biological variable. Fifth, this study is limited to a single side-cutting speed and angle. Consequently, the identified compensatory patterns may not be generalizable to maneuvers performed at different velocities or with different directional changes, as these variables could elicit distinct biomechanical strategies. Finally, lack of electromyographic (EMG) data in this study limits our insight into the underlying neuromuscular control mechanisms.

## 5. Clinical and Rehabilitation Implications

The three-phase compensatory model during side-cutting identified in this study may provide a critical biomechanical basis for refining rehabilitation assessment and training in ACLR patients prior to return to sport. Given that altered movement control patterns and deficits in postural stability during dynamic activities are considered predictors of ACL reinjury [10], consequently, clinicians should implement targeted neuromuscular training after ACLR [46]. Interventions should focus on improving rotational control during deceleration, promoting symmetrical loading during stance, and strengthening the quadriceps for push-off. This approach aims to improve lower-limb biomechanical symmetry and reduce the risks of future knee osteoarthritis [47] and secondary injury.

## 6. Conclusions

This study proposes a three-phase compensatory framework that reveals distinct, sequential deficits during side-cutting: “rotational instability” at initial contact (characterized by an increased external rotation angle), “protective unloading” during stance (characterized by decreased mGRF and vGRF), and “force-generation deficit” at push-off (characterized by reduced aGRF and extension moment). The proposed three-phase model delineates specific biomechanical deficits that can guide targeted rehabilitation protocols and performance optimization following ACLR.

## Figures and Tables

**Table 1:** Participant Characteristics ^a^.

Characteristic	ACLR Group, Mean ± SD/%
Age, y	33.59 ± 6.93
Sex (male/female)	19 (55.88%)/15 (44.12%)
Body mass, kg	75.96 ± 12.19
Body height, cm	173.28 ± 8.07
Concomitant meniscal injury (Yes/No)	9 (26.47%)/25 (73.53%)
Time since surgery, mo	9.45 ± 0.55
Preoperative Tegner score	6.50 ± 1.74
IKDC score	65.34 ± 6.94
Lysholm Knee Score	83.06 ± 12.05

^a^ ACLR: anterior cruciate ligament reconstruction; IKDC: International Knee Documentation Committee Subjective Knee Form; Tegner: Tegner Activity Scale.

**Table 2:** Peak knee angles/moments during the entire stance phase.

Items	Reconstructed Limb Mean ± SD	Contralateral Limb Mean ± SD	*p* Values	Effect Size (Cohen’s d)
Peak values				
Peak knee flexion angle (°)	46.427 ± 6.140	51.560 ± 7.255	<0.001 *	0.85
Peak knee abduction angle (°)	5.268 ± 3.925	5.139 ± 3.582	0.806	NA
Peak knee external rotation angle (°)	10.036 ± 5.726	8.156 ± 4.587	0.037 *	0.37
Peak knee extension moments (BW·BH)	0.122 ± 0.039	0.169 ± 0.036	<0.001 *	1.06
Peak knee abduction moments (BW·BH)	0.026 ± 0.027	0.032 ± 0.029	0.268	NA
Peak knee external rotation moments (BW·BH)	−0.002 ± 0.007	−0.003 ± 0.010	0.712	NA

* *p* < 0.05; NA, not applicable, as effect sizes are not reported for nonsignificant findings (*p* ≥ 0.05); BW·BH, body weight multiplied by body height.

**Table 3:** Summary of Inter-limb Differences During the Three Phases of Side-Cutting.

Phase	Items	Reconstructed Limb Mean ± SD	Contralateral Limb Mean ± SD	*p* Values	Effect Size (Cohen’s d)
Initial contact-deceleration phase	Knee external rotation angle at p_1_GRF peak (°)	8.487 ± 5.882	6.335 ± 5.205	0.021 *	0.42
	Knee extension moments at p_1_GRF peak (BW·BH)	0.053 ± 0.027	0.067 ± 0.030	0.020 *	0.42
	Knee flexion angle at p_2_GRF peak (°)	43.183 ± 5.434	47.263 ± 6.350	<0.001 *	0.81
	Knee extension moments at p_2_GRF peak (BW·BH)	0.101 ± 0.035	0.135 ± 0.031	<0.001 *	0.79
	Knee abduction moments at p_2_GRF peak (BW·BH)	0.026 ± 0.015	0.032 ± 0.017	0.038 *	0.37
Stance pivot phase	Peak mGRF (BW)	0.484 ± 0.129	0.518 ± 0.119	0.029 *	0.39
	Peak vGRF (BW)	1.981 ± 0.282	2.099 ± 0.30	0.012 *	0.46
	Knee flexion angle at mGRF peak (°)	41.889 ± 6.431	46.880 ± 7.163	<0.001 *	0.67
	Knee extension moments at mGRF peak (BW·BH)	0.095 ± 0.036	0.138 ± 0.035	0.037 *	0.96
	Knee flexion angle at vGRF peak (°)	43.362 ± 6.471	48.338 ± 8.343	0.001 *	0.63
	Knee external rotation angle at vGRF peak (°)	4.879 ± 5.372	2.918 ± 4.921	0.036 *	0.37
	Knee extension moments at vGRF peak (BW·BH)	0.111 ± 0.041	0.151 ± 0.039	<0.001 *	0.82
Push-off phase	Peak aGRF (BW)	0.198 ± 0.066	0.226 ± 0.062	0.010 *	0.47
	Knee extension moments at aGRF peak (BW·BH)	0.008 ± 0.013	0.014 ± 0.016	0.037 *	0.37

* *p* < 0.05; p_1_GRF, first posterior ground reaction force; p_2_GRF, second posterior ground reaction force; mGRF, medial ground reaction force; vGRF, vertical ground reaction force; aGRF, anterior ground reaction force; BW, body weight; BW·BH, body weight multiplied by body height.

**Table 4:** Biomechanical parameters of the knee joint during the initial contact-deceleration phase.

Items	Reconstructed Limb Mean ± SD	Contralateral Limb Mean ± SD	*p* Values	Effect Size (Cohen’s d)
p_1_GRF (BW)	0.244 ± 0.123	0.226 ± 0.104	0.430	NA
p_2_GRF (BW)	0.287 ± 0.125	0.306 ± 0.094	0.280	NA
p_1_GRF peak values				
Knee flexion angle (°)	31.838 ± 5.961	32.723 ± 6.530	0.274	NA
Knee abduction angle (°)	1.403 ± 4.280	1.180 ± 3.850	0.669	NA
Knee external rotation angle (°)	8.487 ± 5.882	6.335 ± 5.205	0.021 *	0.42
Knee extension moments (BW·BH)	0.053 ± 0.027	0.067 ± 0.030	0.020 *	0.42
Knee abduction moments (BW·BH)	0.010 ± 0.017	0.008 ± 0.020	0.349	NA
Knee external rotation moments (BW·BH)	0.001 ± 0.002	0.001 ± 0.003	0.658	NA
p_2_GRF peak values				
Knee flexion angle (°)	43.183 ± 5.434	47.263 ± 6.350	<0.001 *	0.81
Knee abduction angle (°)	1.947 ± 4.691	1.460 ± 4.465	0.465	NA
Knee external rotation angle (°)	5.643 ± 5.077	4.024 ± 4.900	0.065	NA
Knee extension moments (BW·BH)	0.101 ± 0.035	0.135 ± 0.031	<0.001 *	0.79
Knee abduction moments (BW·BH)	0.026 ± 0.015	0.032 ± 0.017	0.038 *	0.37
Knee external rotation moments (BW·BH)	0.0001 ± 0.004	0.001 ± 0.003	0.206	NA

* *p* < 0.05; NA, not applicable, as effect sizes are not reported for nonsignificant findings (*p* ≥ 0.05); p_1_GRF, first posterior ground reaction force; p_2_GRF, second posterior ground reaction force; BW, body weight; BW·BH, body weight multiplied by body height.

**Table 5:** Biomechanical parameters of the knee joint during the Stance pivot phase.

Items	Reconstructed Limb Mean ± SD	Contralateral Limb Mean ± SD	*p* Values	Effect Size (Cohen’s d)
Peak mGRF (BW)	0.484 ± 0.129	0.518 ± 0.119	0.029 *	0.39
Peak vGRF (BW)	1.981 ± 0.282	2.099 ± 0.30	0.012 *	0.46
mGRF peak values				
Knee flexion angle (°)	41.889 ± 6.431	46.880 ± 7.163	<0.001 *	0.67
Knee abduction angle (°)	3.719 ± 4.840	3.332 ± 4.582	0.565	NA
Knee external rotation angle (°)	3.984 ± 4.839	2.292 ± 4.919	0.056	NA
Knee extension moments (BW·BH)	0.095 ± 0.036	0.138 ± 0.035	0.037 *	0.96
Knee abduction moments (BW·BH)	0.020 ± 0.018	0.023 ± 0.023	0.500	NA
Knee external rotation moments (BW·BH)	0.002 ± 0.006	0.003 ± 0.008	0.558	NA
vGRF peak values				
Knee flexion angle (°)	43.362 ± 6.471	48.338 ± 8.343	0.001 *	0.63
Knee abduction angle (°)	3.072 ± 4.924	2.931 ± 4.581	0.846	NA
Knee external rotation angle (°)	4.879 ± 5.372	2.918 ± 4.921	0.036 *	0.37
Knee extension moments (BW·BH)	0.111 ± 0.041	0.151 ± 0.039	<0.001 *	0.82
Knee abduction moments (BW·BH)	0.022 ± 0.018	0.025 ± 0.022	0.444	NA
Knee external rotation moments (BW·BH)	0.002 ± 0.006	0.003 ± 0.007	0.590	NA

* *p* < 0.05; NA, not applicable, as effect sizes are not reported for nonsignificant findings (*p* ≥ 0.05); mGRF, medial ground reaction force; vGRF, vertical ground reaction force; BW, body weight; BW·BH, body weight multiplied by body height.

**Table 6:** Biomechanical parameters of the knee joint during the Push-off phase.

Items	Reconstructed Limb Mean ± SD	Contralateral Limb Mean ± SD	*p* Values	Effect Size (Cohen’s d)
Peak aGRF (BW)	0.198 ± 0.066	0.226 ± 0.062	0.010 *	0.47
aGRF peak values				
Knee flexion angle (°)	26.522 ± 6.180	26.780 ± 6.091	0.824	NA
Knee abduction angle (°)	3.197 ± 3.905	3.241 ± 3.401	0.929	NA
Knee external rotation angle (°)	5.325 ± 5.039	3.858 ± 4.402	0.086	NA
Knee extension moments (BW·BH)	0.008 ± 0.013	0.014 ± 0.016	0.037 *	0.37
Knee abduction moments (BW·BH)	0.003 ± 0.009	0.007 ± 0.012	0.055	NA
Knee external rotation moments (BW·BH)	0.0003 ± 0.003	0.001 ±0.003	0.153	NA

* *p* < 0.05; NA, not applicable, as effect sizes are not reported for nonsignificant findings (*p* ≥ 0.05); aGRF, anterior ground reaction force; BW, body weight; BW·BH, body weight multiplied by body height.

## Data Availability

The original contributions presented in the study are included in the article, further inquiries can be directed to the corresponding authors.

## Abbreviations

The following abbreviations are used in this manuscript:

ACLR	anterior cruciate ligament reconstruction
GRF	ground reaction force
p_1_GRF	first posterior ground reaction force
p_2_GRF	second posterior ground reaction force
vGRF	vertical ground reaction force
mGRF	medial ground reaction force
aGRF	anterior ground reaction force
BW	body weight
BH	body height
IKDC	International Knee Documentation Committee Subjective Knee Form
Tegner	Tegner Activity Scale
BW·BH	body weight multiplied by body height
